# Supplementary material for: Atlantic-origin water extension into the Pacific Arctic induced an anomalous biogeochemical event
Source: Nat Commun. 2023 Nov 2;14:6235. doi: 10.1038/s41467-023-41960-w (PMC10622542; doi:10.1038/s41467-023-41960-w)
Supplement: Supplementary file 1 — Supplementary Information [file 41467_2023_41960_MOESM1_ESM.pdf]

Supplementary Information for

Atlantic-origin water extension into the Pacific Arctic induced an anomalous biogeochemical event

Shigeto Nishino[1]*†, Jinyoung Jung[2]†, Kyoung-Ho Cho[2], William J. Williams[3], Amane Fujiwara[1], Akihiko Murata[4], Motoyo Itoh[1], Eiji Watanabe[1], Michio Aoyama[4,5], Michiyo Yamamoto-Kawai[6], Takashi Kikuchi[1], Eun Jin Yang[2] & Sung-Ho Kang[7]

[1]Institute of Arctic Climate and Environment Research, Research Institute for Global Change, Japan Agency for Marine-Earth Science and Technology (JAMSTEC); Yokosuka, 237-0061, Japan.

[2]Division of Ocean Sciences, Korea Polar Research Institute; Incheon, 21990, Republic of Korea.

[3]Institute of Ocean Sciences, Fisheries and Oceans Canada; Sidney, BC, V8L 4B2, Canada.

[4]Global Ocean Observation Research Center, Research Institute for Global Change, Japan Agency for Marine-Earth Science and Technology (JAMSTEC); Yokosuka, 237-0061, Japan.

[5]Center for Research in Isotopes and Environmental Dynamics, University of Tsukuba; Tsukuba, 305-8577, Japan.

[6]Department of Ocean Sciences, Tokyo University of Marine Science and Technology; Minato-ku, Tokyo, 108-8477, Japan.

[7]Korea Polar Research Institute; Incheon, 21990, Republic of Korea.

*Corresponding author. Email: nishinos@jamstec.go.jp

†These authors contributed equally: Shigeto Nishino, Jinyoung Jung

This file includes:

Supplementary Discussion 1: Source of the anomalous water on the Chukchi Plateau

Supplementary Discussion 2: Interpolation uncertainties

Supplementary Discussion 3: Vertical mixing between the Lower Halocline Water layer and oxygen saturation minimum layer

Supplementary Figs. 1–7

Supplementary Table 1

Supplementary References

Supplementary Discussion 1: Source of the anomalous water on the Chukchi Plateau

Here, we compare vertical sections of hydrographic and biogeochemical parameters across two seas, the Chukchi and East Siberian seas, to identify the source of anomalously low oxygen saturation (dissolved oxygen) and acidified water found on the Chukchi Plateau (CP) in 2020 (Fig. 3). The vertical section from the Chukchi shelf-slope to the southwestern side of the CP (Supplementary Fig. 1a) shows that the anomalously low oxygen saturation water appeared on the seafloor north of 74° N. The low oxygen saturation corresponded with low NO (Supplementary Fig. 1b), where NO is defined as $9[NO_3^-] + [O_2]$ (μmol kg^{-1}) and used as a quasi-conservative tracer that is independent of biological processes[1]. Thus, the low oxygen saturation and low NO water are assumed to be delivered from a remote region rather than formed by local microbial oxygen consumption.

Low oxygen saturation and acidified water were found on the East Siberian Sea (ESS) shelf with salinity of about 30–32 and on the shelf-slope where salinity is 34–34.5 (Supplementary Fig. 2a, b) (ref. [2]). The low oxygen saturation and acidified water on the shelf-slope could be formed by brine rejection in the ESS shelf[3] and/or through a long-time contact of the water with the shelf-slope sediments[4]. The low oxygen saturation and acidified water in the ESS are also characterized by low NO, which is likely caused by the input of river water containing low preformed nitrate, sedimentary denitrification, and decomposition of terrestrial organic matters that are relatively nitrogen-poor compared to marine organic matters[5]. In addition, the ratio NO/PO, where PO is another quasi-conservative tracer defined as $135[PO_4^{3-}] + [O_2]$ (μmol kg^{-1})[1], likely has a value less than 0.75 in the ESS, which is the lowest value among the pan-Arctic seas[6]. Likewise, ESS shelf-slope water was characterized by low NO values (200–300 μmol kg^{-1}; Supplementary Fig. 2c) and NO/PO $<$ 0.75 (Supplementary Fig. 2d). The anomalously low oxygen saturation and acidified water beside the CP were also low in NO (~230 μmol kg^{-1}; Supplementary Fig. 1b) and NO/PO = ~0.66 (not shown). Therefore, ESS shelf-slope water with salinity = 34–34.5, NO = 200–300 μmol kg^{-1}, and NO/PO $<$ 0.75 was probably transported to the CP, on which we found water with similar characteristics (anomalously low oxygen saturation and acidified water with salinity = ~34.5, NO = ~230 μmol kg^{-1}, and NO/PO = ~0.66).

Supplementary Discussion 2: Interpolation uncertainties

We created gridded datasets using an optimal interpolation method with interpolation uncertainties (see Methods). Uncertainties in dynamic height (Supplementary Fig. 3a–c) were large in the southern branch of the Beaufort Gyre (BG) over the Chukchi shelf-slope, where many eddies were noted to emerge[7,8]. Due to the presence of eddies, the dynamic height varied in space and time, resulting in significant interpolation uncertainties. These uncertainties were relatively small in the 1950s–1980s because the slow BG circulation and its accompanying eddy activity was weak. In 2017–2020, the uncertainty was relatively large in the northward flow along ~170° W, located on the western side of the CP. This northward flow occurred at a front between Pacific Water (PW) and Lower Halocline Water (LHW) caused by the penetration of LHW. The penetration of LHW is shown as a depression of the dynamic height in 2017–2020 compared with that in 2008–2016 west of the CP (Supplementary Fig. 4a). In this area, the depression was larger than the interpolation uncertainty. Hence, we can safely mention that the LHW penetration occurred in 2017–2020, accompanied by the formation of frontal northward flow.

Uncertainties in the vertical salinity sections (Supplementary Fig. 3d–f) were significant in the halocline, where salinity increases sharply with depths at the surface and in an intermediate layer with salinity between 33 and 34. Except for the surface, the uncertainty was largest in the halocline above the CP in 2017–2020 because the front between the PW and LHW was formed and fluctuated around the CP. Increasing salinity, which eventually became larger than the uncertainty, appeared at depths of 100–150 m west of the CP in 2017–2020 (Supplementary Fig. 4b). This salinity increase was caused by shoaling of the halocline, consistent with the LHW penetration to the west of the CP in 2017–2020.

Uncertainties in oxygen saturation (Supplementary Fig. 3g–i) were prominent at the locations with the lowest oxygen saturation in each period. In 2008–2016 and the 1950s–1980s, the oxygen saturation was lowest along the ESS shelf-slope, increasing sharply toward the north. This significant spatial variation in oxygen saturation resulted in large interpolation uncertainties. On the contrary, in 2017–2020, oxygen saturation was the lowest, and the uncertainty was the largest along ~170° W. During this period, water with anomalously low oxygen saturation was only identified on the CP (near 170° W) in 2020. This temporal variation in oxygen saturation caused an enlargement of the uncertainty at that location. Differences in oxygen saturation between 2017–2020 and 2008–2016 indicate that the oxygen saturation increased along the ESS shelf-slope and decreased along ~170° W (Supplementary Fig. 4c). The decrease in oxygen saturation along ~170° W was smaller than the uncertainty associated with the large temporal variation in oxygen saturation.

Supplementary Discussion 3: Vertical mixing between the Lower Halocline Water layer and oxygen saturation minimum layer

A possible reason for the lower levels of oxygen saturation in the LHW layer of the Canada Basin (CB) in 2017–2020 (Fig. 5g) compared to 2008–2016 (Fig. 5h) is thought to be vertical mixing between the LHW layer (salinity = 34–34.5) and the above layer characterized by a vertical minimum of oxygen saturation. The layer of the oxygen saturation minimum was thicker and the minimum values were lower in 2017–2020 (Supplementary Fig. 5a) compared to 2008–2016 (Supplementary Fig. 5b). This may be attributed to sedimentation processes such as increases in export production[9] and coastal erosion[10] due to the recent sea ice loss. Evidence of the vertical mixing is shown in the vertical section of NO, where NO is a quasi-conservative tracer[1] and its vertical minimum is identified with LHW[11]. In 2017–2020, the vertical mixing would increase the NO in the LHW layer toward the south, that is, toward the downstream of the southward flow of the BG (Supplementary Fig. 5c). On the other hand, in 2008–2016, the NO increase in the LHW layer toward the south was small except for the shelf-slope region (Supplementary Fig. 5d), suggesting weak vertical mixing that could not contribute to decreasing the oxygen saturation in the LHW layer (Supplementary Fig. 5b) as much as its decrease observed in the CB in 2017–2020 (Supplementary Fig. 5a).

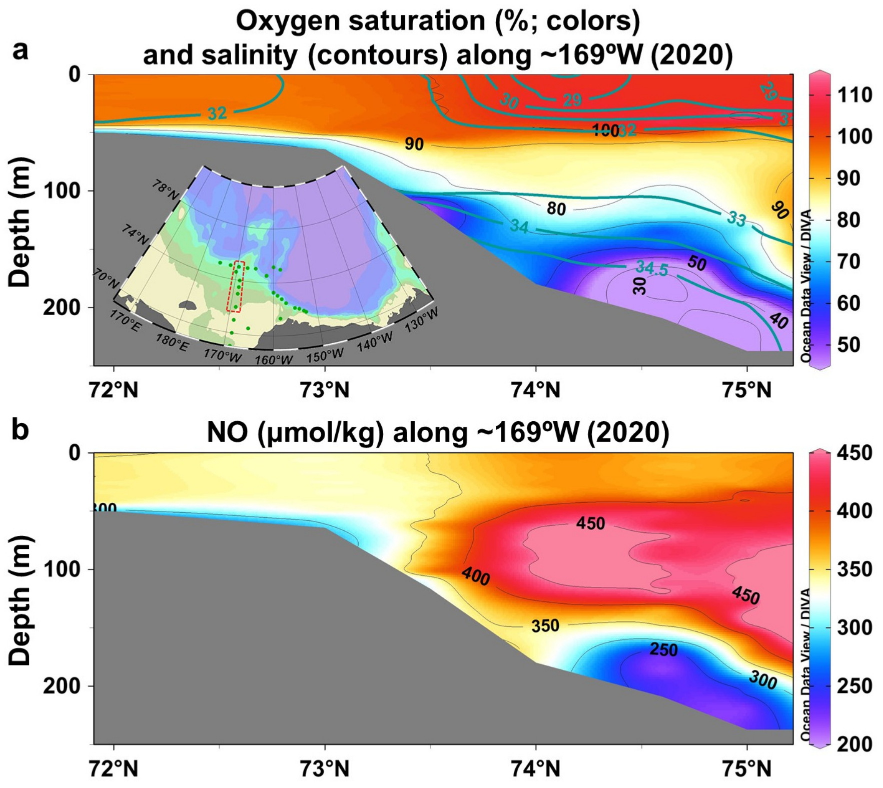

Supplementary Fig. 1 | Water characteristics from Chukchi shelf-slope to the southwestern side of Chukchi Plateau in 2020. Vertical sections of (**a**) oxygen saturation (%) and (**b**) NO (µmol kg^{-1}) along ~169° W in 2020. In (**a**), salinity contours are drawn as green lines. The data were obtained from sensors attached to the CTD system in the 2020 Research Vessel Mirai cruise. For the illustrations of vertical sections, we used the data in an area enclosed by a red square in the embedded map in (**a**).

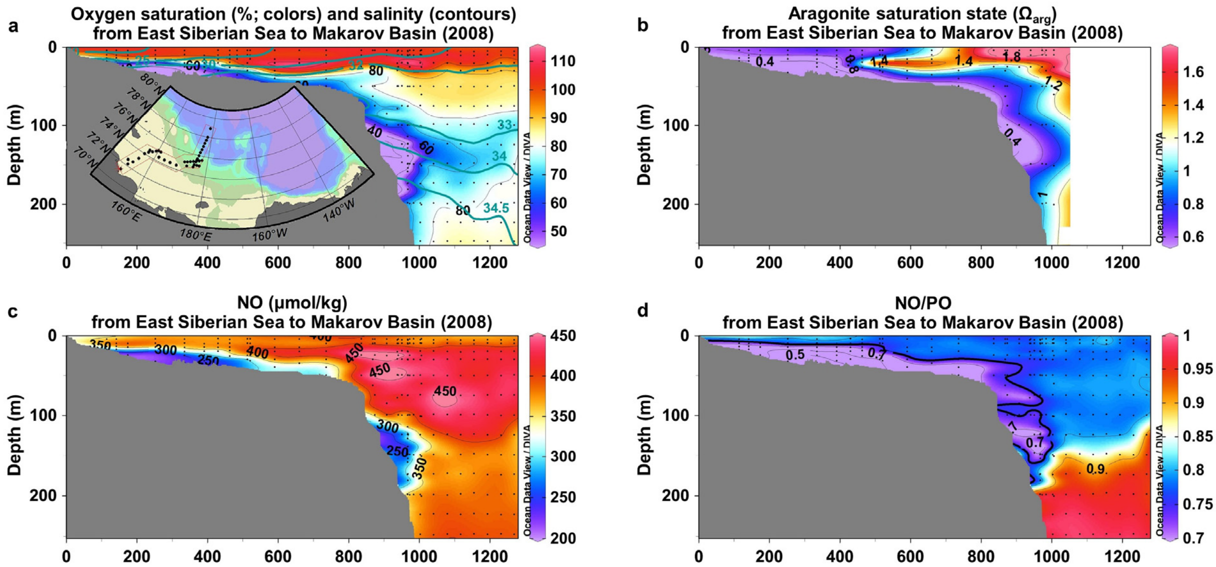

Supplementary Fig. 2 | Water characteristics from Eastern Siberian Sea to Makarov Basin in 2008. Vertical sections of (**a**) oxygen saturation (%), (**b**) aragonite saturation state (Ω_{arg}), (**c**) NO (μmol kg^{-1}), and (**d**) NO/PO along a line in the embedded map in (**a**), which corresponds to the horizontal axis (km) in each section. Black dots indicate the data points. In (**a**), salinity contours are drawn as green lines. In (**d**), the contour of 0.75 is shown by a thick curve. The data were obtained from the International Siberian Shelf Study 2008 and the 2008 Research Vessel Mirai cruise.

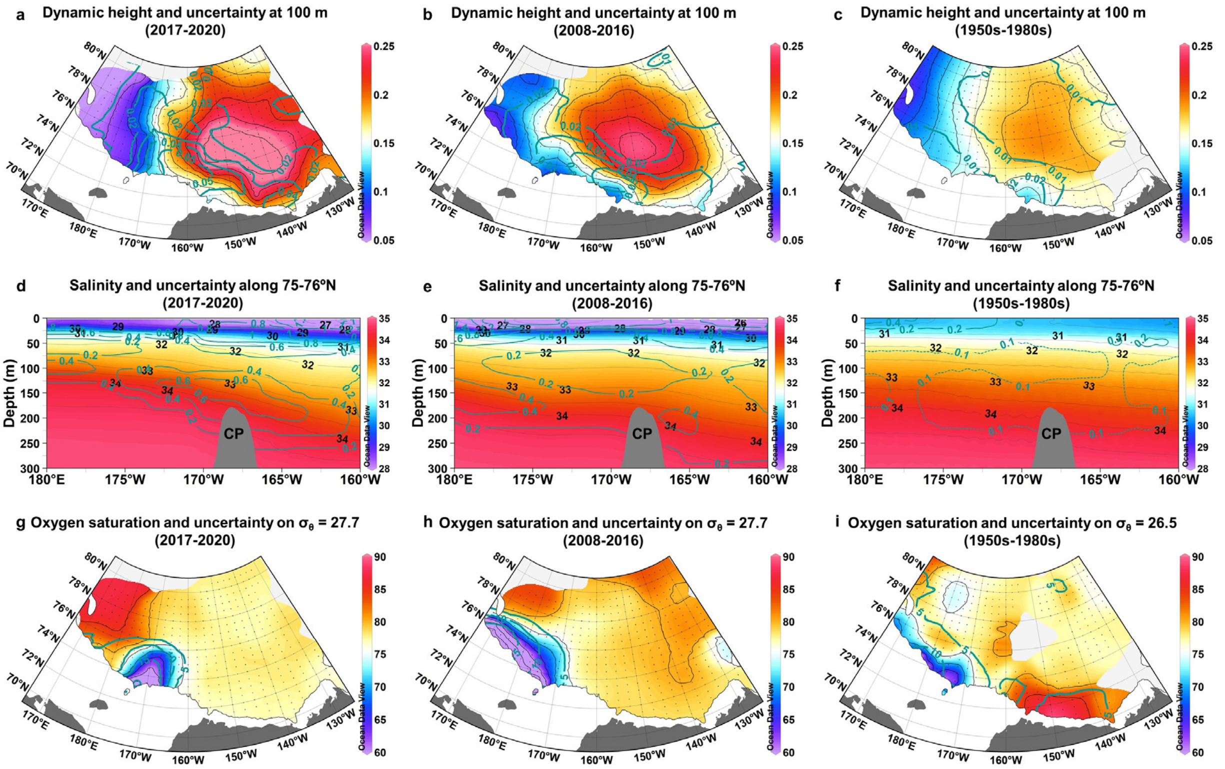

Supplementary Fig. 3 | Uncertainties of the gridded data. a–c Dynamic height (dyn m; colors) at 100 m relative to 250 m and uncertainty (green contours) in (**a**) 2017–2020, (**b**) 2008–2016, and (**c**) the 1950s–1980s. Black dots represent 0.5° × 2.5° latitude–longitude gridded points where interpolation values and uncertainties can be calculated using the data obtained from the cruises listed in Supplementary Table 1 and historical data (see Methods). **d–f** Vertical sections of salinity (colors) and uncertainty (green contours) along a band of 75–76° N (blue dashed square in Fig. 5a–c) with a 1.0° longitude grid in (**d**) 2017–2020, (**e**) 2008–2016, and (**f**) the 1950s–1980s. Chukchi Plateau is abbreviated as CP. **g–i** Oxygen saturation (%; colors) and uncertainty (green contours) along isopycnal surfaces of (**g**) σ_θ = 27.7 in 2017–2020, (**h**) σ_θ = 27.7 in 2008–2016, and (**i**) σ_θ = 26.5 in the 1950s–1980s. Black dots indicate 0.5° × 2.5° latitude–longitude gridded points where interpolation values and uncertainties can be calculated.

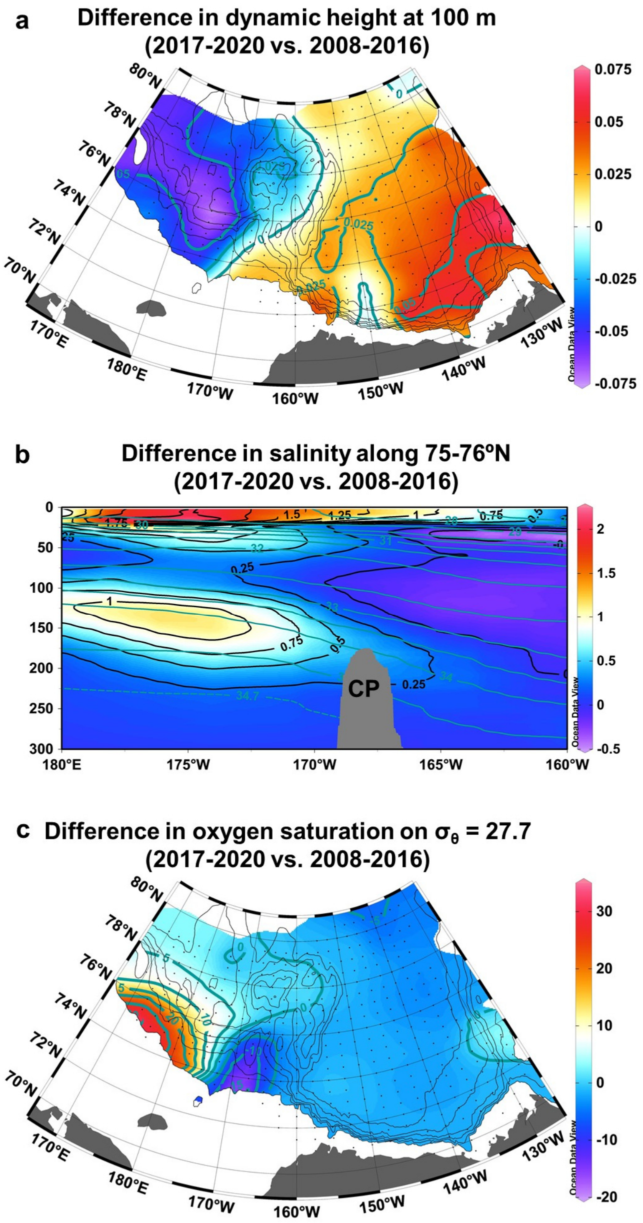

Supplementary Fig. 4 | Differences of the gridded data between 2017–2020 and 2008–2016. a Difference in dynamic height (dyn m) at 100 m relative to 250 m between 2017–2020 and 2008–2016. Black dots represent 0.5° × 2.5° latitude–longitude gridded points where interpolation values can be calculated in 2017–2020 and 2008–2016. **b** Vertical section showing the difference in salinity between 2017–2020 and 2008–2016 along a band of 75–76° N (blue dashed square in Fig. 5a, b) with a 1.0° longitude grid. Salinity contours in 2017–2020 are overlaid. Chukchi Plateau is abbreviated as CP. **c** Difference in oxygen saturation (%) on the isopycnal surface of σ_θ = 27.7 between 2017–2020 and 2008–2016. Black dots indicate 0.5° × 2.5° latitude–longitude gridded points where interpolation values can be calculated in 2017–2020 and 2008–2016.

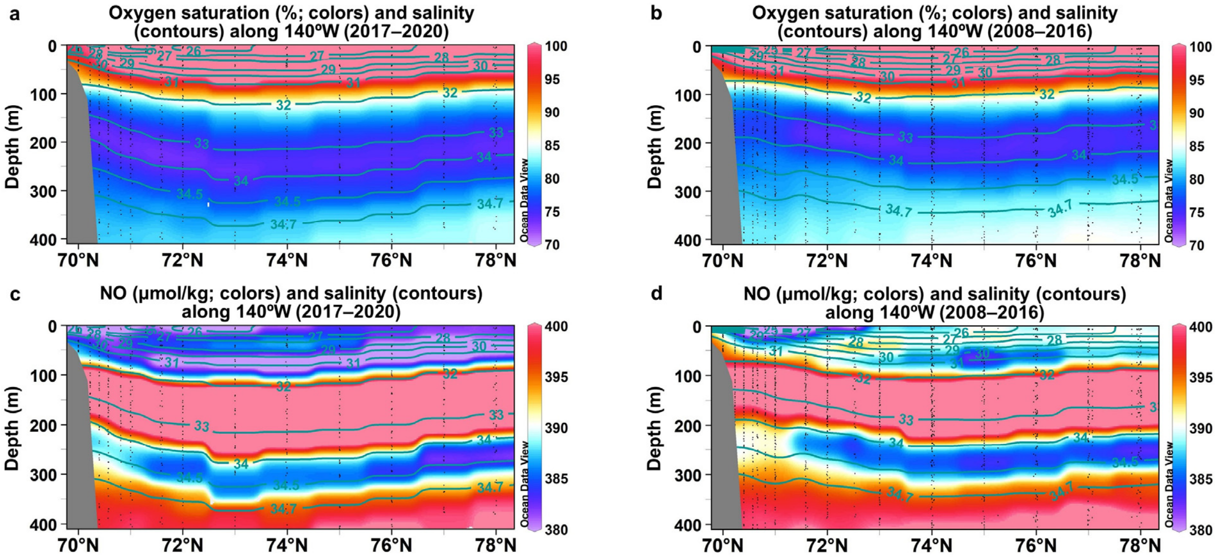

Supplementary Fig. 5 | Water characteristics along 140° W across Canada Basin in 2017–2020 and 2008–2016. a, **b** Vertical sections of oxygen saturation (%; colors) and salinity (contours) along 140° W in (**a**) 2017–2020 and (**b**) 2008–2016. **c**, **d** Vertical sections of NO (μmol kg^{-1}; colors) and salinity (contours) along 140° W in (**c**) 2017–2020 and (**d**) 2008–2016. Black dots indicate the data points in each panel. The data were obtained from the Canada/US Beaufort Gyre Exploration Project by Canadian Coast Guard Ship Louis S. St-Laurent between 2008 and 2020.

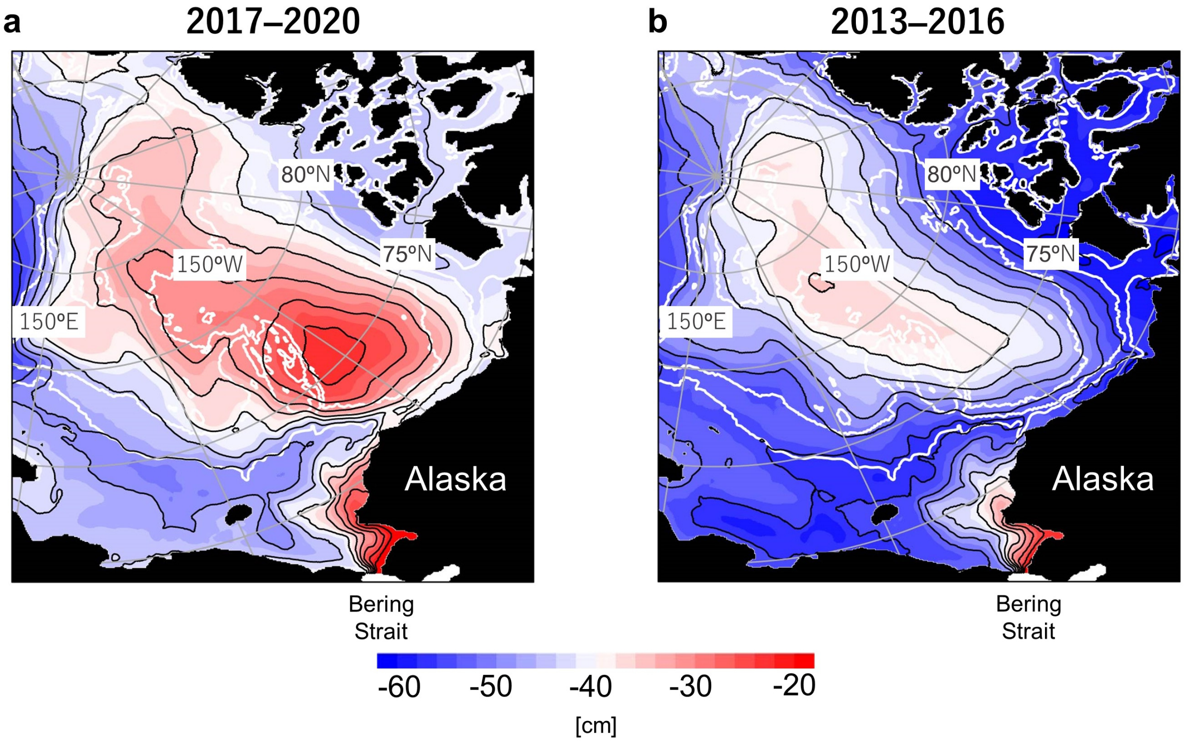

Supplementary Fig. 6 | Simulated surface ocean circulation. Sea surface height (cm) averaged for (**a**) 2017–2020 and (**b**) 2013–2016, which was simulated using a physical sea ice–ocean general circulation model (see Methods).

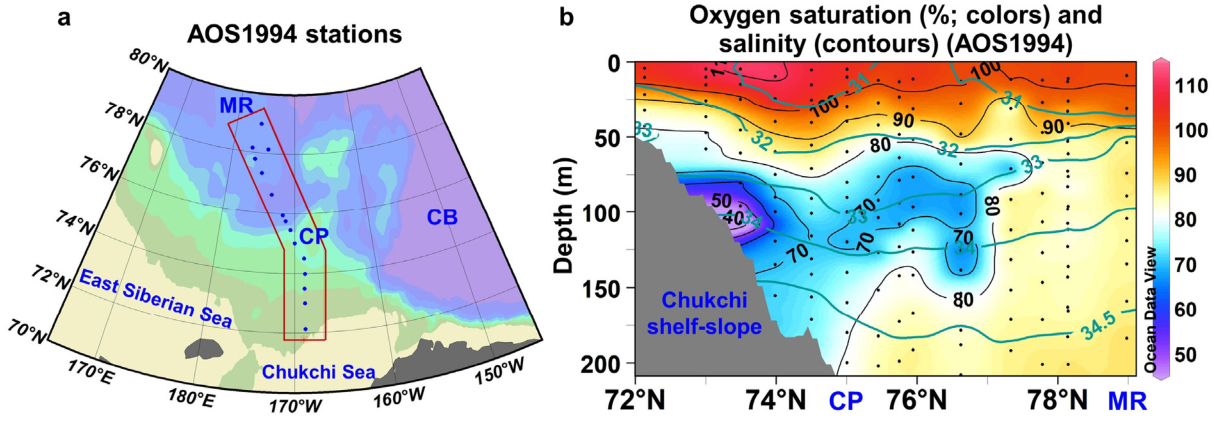

Supplementary Fig. 7 | Water characteristics from Chukchi shelf-slope to Mendeleyev Ridge via Chukchi Plateau in 1994. a Stations (blue dots) of the Arctic Ocean Section (AOS) in 1994 by Canadian Coast Guard Ship Louis S. St-Laurent. Geographical locations are abbreviated as follows: Canada Basin (CB), Chukchi Plateau (CP), and Mendeleyev Ridge (MR). **b** Vertical section of oxygen saturation (%; colors) and salinity (contours) along a red square in (**a**). Black dots indicate the data points.

Supplementary Table 1 | Expeditions, observation periods, and data sites.

Expeditions	Observation periods	Data sites
Araon 2020	August 4 – August 31, 2020	https://kpdc.kopri.re.kr/search/694ee19e-1c0b-4a44-8fd6-e83c94992731
Mirai 2002	September 2 – October 10, 2002	
2004	September 1 – October 12, 2004	
2008	August 26 – October 9, 2008	
2009	September 7 – October 15, 2009	
2010	September 2 – October 16, 2010	
2012	September 3 – October 17, 2012	
2013	August 28 – October 21, 2013	
2014	August 31 – October 10, 2014	http://www.godac.jamstec.go.jp/darwin/e
2015	August 24 – October 22, 2015	
2016	August 22 – October 5, 2016	
2017	August 23 – October 1, 2017	
2018	October 24 – December 7, 2018	
2019	September 28 – November 10, 2019	
2020	September 19 – November 2, 2020	
BGEP 2003	August 7 – September 7, 2003	
2004	July 29 – September 2, 2004	
2005	July 29 – September 1, 2005	
2006	August 5–- September 14, 2006	
2007	July 26 – August 31, 2007	
2008	July 17 – August 21, 2008	
2009	September 17 – October 15, 2009	
2010	September 15 – October 15, 2010	
2011	July 21 – August 18, 2011	https://www2.whoi.edu/site/beaufortgyre/data/ctd-and-geochemistry/
2012	August 2 – September 8, 2012	
2013	August 1 – September 2, 2013	
2014	September 21 – October 17, 2014	
2015	September 20 – October 16, 2015	
2016	September 22 – October 18, 2016	
2017	September 7 – October 2, 2017	
2018	September 7 – October 2, 2018	
2019	September 12 – October 4, 2019	
2020	September 14 – October 2, 2020	
AOS 1994	July 24 – September 9, 1994	https://cchdo.ucsd.edu/cruise/18SN19940724
CBL 2002	August 19 – September 23, 2002	http://psc.apl.washington.edu/HLD/CBL/CBL.html
ISSS 2008	August 15 – September 26, 2008	https://cchdo.ucsd.edu/cruise/90JS20080815

The expeditions conducted by the Research Vessel (R/V) Araon (Korea) and R/V Mirai (Japan) are expressed as Araon and Mirai, respectively, with the expedition years. The Canada/US Beaufort Gyre Exploration Project using Canadian Coast Guard Ship (CCGS) Louis S. St-Laurent

is abbreviated as BGEP. The 1994 Arctic Ocean Section implemented by the CCGS Louis S. St-Laurent is expressed as AOS 1994. The Chukchi Borderland Project and International Siberian Shelf Study carried out by the United States Coast Guard Cutter Polar Star (USA) in 2002 and Yacob Smirniskyi (Russia) in 2008, respectively, are presented as CBL 2002 and ISSS 2008.

Supplementary References

1. Broecker, W. S. "NO", a conservative water-mass tracer. *Earth Planet. Sci. Lett.* **23**, 100–107 (1974). https://doi.org/10.1016/0012-821X(74)90036-3
2. Cross, J. N., Mathis, J. T., Pickart, R. S. & Bates, N. R. Formation and transport of corrosive water in the Pacific Arctic region. *Deep-Sea Res. II* **152**, 67–81 (2018). https://doi.org/10.1016/j.dsr2.2018.05.020
3. Anderson, L. G., Andersson, P. S., Björk, G., Jones, E. P., Jutterström, S. & Wåhlström, I. Source and formation of the upper halocline of the Arctic Ocean. *J. Geophys. Res. Oceans* **118**, 410–421 (2013). https://doi.org/10.1029/2012JC008291
4. Anderson, L. G. et al. Shelf–Basin interaction along the East Siberian Sea. *Ocean Sci.* **13**, 349–363 (2017). https://doi.org/10.5194/os-13-349-2017
5. Alkire, M. B., Rember, R. & Polyakov, I. Discrepancy in the identification of the Atlantic/Pacific front in the central Arctic Ocean: NO versus nutrient relationships. *Geophys. Res. Lett.* **46**, 3843–3852 (2019). https://doi.org/10.1029/2018GL081837
6. Wilson, C. & Wallace, D. W. R. Using the nutrient ratio NO/PO as a tracer of continental shelf waters in the central Arctic Ocean. *J. Geophys. Res.* **95**, 22,193–22,208 (1990). https://doi.org/10.1029/JC095iC12p22193
7. Zhao, M., Timmermans, M.-L., Cole, S., Krishfield, R. & Toole, J. Evolution of the eddy field in the Arctic Ocean's Canada Basin, 2005–2015. *Geophys. Res. Lett.* **43**, 8106–8114 (2016). https://doi.org/10.1002/2016GL069671
8. Meneghello, G., Marshall, J., Cole, S. T. & Timmermans, M.-L. Observational inferences of lateral eddy diffusivity in the halocline of the Beaufort Gyre. *Geophys. Res Lett.* **44**, 12,331–12,338 (2017). https://doi.org/10.1002/2017GL075126
9. Lewis, K. M., Van Dijken, G. L. & Arrigo, K. R. Changes in phytoplankton concentration now drive increased Arctic Ocean primary production. *Science* **369**, 198-202 (2020). https://doi.org/10.1126/science.aay8380
10. Semiletov, I. et al. Acidification of East Siberian Arctic Shelf waters through addition of freshwater and terrestrial carbon. *Nat. Geosci.* **9**, 361–367 (2016). https://doi.org/10.1038/NEGO2695
11. Jones, E. P. & Anderson, L. G. On the origin of the chemical properties of the Arctic Ocean halocline. *J. Geophys. Res.* **91**, 10759–10767 (1986). https://doi.org/10.1029/JC091iC09p10759
