# Supplementary material for: Atlantic-origin water extension into the Pacific Arctic induced an anomalous biogeochemical event
Source: Nat Commun. 2023 Nov 2;14:6235. doi: 10.1038/s41467-023-41960-w (PMC10622542; doi:10.1038/s41467-023-41960-w)
Supplement: Supplementary file 3 — Description of Additional Supplementary Files [file 41467_2023_41960_MOESM3_ESM.docx]

**Description of Additional Supplementary Files**

**File Name: Supplementary Data 1**

**Description:** Research Vessel Mirai data from CTD and bottle samples. The data are text files separated by the CTD and bottle for each year (e.g., Mirai_CTD_2020.txt and Mirai_Btl_2020.txt). Once the issues related to a security incident have been resolved, the data can be retrieved from the Research Vessel Mirai data site shown in Supplementary Table 1.
